# Cases of Fractures

**Published:** 1856-11

**Authors:** Frank Hastings Hamilton

**Affiliations:** Buffalo, N. Y.


### BUFFALO MEDICAL JOURNAL

AND

### MONTHLY REVIEW.

VOL. 12. NOVEMBER, 1856. NO. 6.

### ORIGINAL COMMUNICATIONS.

ART. I.—*Cases of Fractures.* Reported by FRANK HASTINGS HAMILTON, M. D., Buffalo, N. Y.

(Continued from Page 290.)

### FRACTURES OF THE FEMUR.—UPPER THIRD.

#### NECK.

CASE I.—*Simple fracture. Result imperfect.*

James Barry, æt. 52. Simple fracture of the femur through the neck. Drs. Morris and Lewis in attendance. They used a straight splint three weeks, and then permitted him to go about on crutches.

I examined the limb three months after the accident occurred, and found it shortened one inch and a half.

CASE II.—*Simple fracture. Result imperfect.*

Dec. 12, 1850, Thomas Kellogg, æt. 77, of Bloomfield, N. Y., fell through the hold of a steamer, at Buffalo, striking partly upon the right trocanter, and bruising also other parts of his body severely.

Dr. Wilcox was called, and on the following day, Dr. Camp, by request of Dr. Wilcox, was added to the counsel. They noticed that the flesh and skin were bruised over the right trocanter, and apprehended a fracture of the neck of the femur, but after a very careful examination they could not discover any satisfactory evidence of such a fracture. The limb was not shortened, nor were the toes turned either outward or inward, unnaturally; but whenever attempts were made to flex the limb it was very painful, and the limb moved spasmodically.

After about two weeks Dr. Wilcox discovered that the limb was shortened. Dr. Camp had seen the patient three or four times with Dr. Wilcox, and neither of these gentlemen found any shortening until now. A straight splint was immediately applied, with extension and counter-extension.

From that time until the period of my examination, about six months after the accident, it continued to shorten. He could notice a marked difference during the last month. Dr. Wilcox was present with me at this examination. The limb measured just three inches less in length than the other; the motions at the joint, or at the seat of fracture, were tolerable free; his toes were neither inclined outward nor inward, unnaturally. The trocanter was not much flattened, and seemed to move on a long radius when the thigh was rotated. He used crutches, and bore no weight on the limb in walking. The thigh seemed to give way, or "spring," when he settled his body upon the limb.

Mr. Kellogg sued the owners of the vessel for damages, but was non-suited on the ground of some technical irregularity in the proceedings.

Case III.—*Simple Fracture. Result imperfect.*

Eddy, of Potter's Corners, Erie Co., N. Y., æt. 78, fell, July 28, 1847, striking on the trocanter major. He fell about four feet.

Dr. Nott in attendance. Dr. Nott laid his limb over a double inclined plane.

I was requested to see him in consultation, thirty-six hours after the accident. The leg was everted, and any attempts to rotate it in the opposite direction, were very painful. I could not detect crepitus. The trocanter was carried upward and backward, and the limb shortened one inch.

I recommended a continuance of the treatment for the present. Subsequently a straight splint was employed, but only for a short time.

I have never seen Mr. Eddy since, but I have learned from Dr. Nott that he was able to get upon crutches in about eight weeks, and that during the four or five years in which he continued to reside in this county, he used a cane, and walked with a manifest halt. Precisely how much the limb was shortened he was unable to say, yet he knew it was shortened.

CASE IV.—*Simple fracture. Result imperfect.*

Mrs. Jane Stewart, of Buffalo, æt. 73, fell July 4th, 1847, while descending a flight of steps, striking upon the trocanter major of the right side.

Dr. Bryant Burwell in attendance. Dr. Burwell requested me to see Mrs. S. on the twenty-ninth day after the accident. No splint had been applied. The limb was shortened half an inch. The leg was everted, but could be easily rotated inward. No crepitus. The principal pain which she suffered was below the trocanter major. The whole limb was œdematous. She had also incontinence of urine. Dr. B. had been obliged to give her opium freely to alleviate her pains.

No change in the treatment was recommended.

During the first three or four months she was unable to lie down, and for about one year she was unable to walk. From this time she gradually recovered her health and strength, and walked with considerable ease, but with a halt. She survived the accident five years, her death having no connection with this injury. The limb was never measured after I saw her.

CASE V.—*Simple fracture. Result imperfect.*

Mrs. Wright, a German woman living in Buffalo, æt. 66, broke the right femur through its neck, by a fall upon the ice, Jan. 25, 1850. She was admitted to the hospital on the same day.

I could not detect any crepitus, nor was there any shortening of the limb, the toes were turned out so as to lie flat upon the bed. We suspected a fracture of the neck of the femur. The patient was laid in bed upon her back, and pillows were placed under her knees.

Feb. 1. We found the leg shortened, and we laid it over a double inclined plane. On the 10th of February she had bed sores, and the splint was removed, the limb being placed again over pillows.

This treatment was continued until the 28th of Feb., when she left the hospital, and I have not seen her since. Shortly before she left we found the thigh shortened half an inch.

CASE VI.— *Simple fracture. Result imperfect.*

Mrs. Catherine Brenoe, æt. 50, broke her right thigh through its neck, either in Feb. or March, of 1852. She was standing upon a box, three feet high, when she fell, striking upon her right hip.

Dr. Hill was called. She was laid upon a bed, but no splints were applied.

July 20, 1852, she was admitted to the Buffalo Hospital of the Sisters of Charity. Dr. Winne's service.

Oct. 1, 1853, I found this woman in my ward. During all of my previous service she had been in a private ward and my attention had not been called to her. She had had no surgical treatment beyond confinement in bed, and this, I understand, was voluntary. Her thigh was shortened one inch and a half, and rotated outward. The trocanter major rotated on a very short axis. There was also a kind of klick, or crepitus, at this point. It hurt her very much to abduct the thigh.

I directed that she should be got out of bed into a chair. This, with great labor, was soon accomplished; but after a few months I found her again lying in bed, and I could never again persuade her to get up. There was no necessity for this, I think, beyond her own strong disinclination to do otherwise.

Nov. 1, 1856. Nearly five years after the accident, she still occupies the same bed, yet her health is tolerably good, and no sufficient reason exists why she should not walk about or sit up.

CASE VII.— *Simple fracture. Result imperfect.*

Mrs. Catherine Langen, æt. 84, while carrying a bundle of wood, her foot slipped and she fell, striking on the left trocanter. She was admitted to the hospital on the following day, (Jan., 1856.) Dr. John Boardman, hospital surgeon, in attendance.

Dr. Boardman could not discover either crepitus or shortening, but the foot and knee were everted. She was directed to lie still in bed. Subsequently Dr. Boardman thought that he detected crepitus, but I was unable to do so at any time.

She left the hospital, Aug. 31, 1856, about eight months after her admission, unable to walk except with crutches. The thigh is shortened half an inch; toes not everted. The trocanter major feels broad and flattened, and the upper part seems to be thrown inward toward the body. I now suspect that this was a fracture of the neck close to the trocanter, and involving the trocanter itself; and also that it is a case of impaction of the neck. The great breadth of the trocanter is probably due to an abundant ossific deposit. I believe it has united by bone.

CASE VIII.— *Simple fracture. Result imperfect.*

James McLaren, æt. 65. Admitted to the Buffalo Hospital, Sept. 8th, 1855, with a fracture of the left thigh bone through the neck. His account of his injury is, that on the 28th of August, he was struck violently on the inside of his left thigh, near its upper end. The blow was, no doubt, sufficient to break the thigh bone, but it is difficult to understand how it should have been broken through its neck by a blow given in that direction.

He was at first seen by Dr. Hill, who called it a fracture of the neck of the femur. I do not know what treatment he adopted. When he became an inmate of the hospital, Dr. Smith laid him in a modification of Gibson's splint.

Oct. 1, 1855, I found him still lying within the same apparatus, but without extension or counter-extension being made. The thigh was shortened three quarters of an inch. I directed him to get up and use crutches. No dressings were subsequently applied.

March 31, 1856. He was still in the hospital walking with a cane, and with some halting in his gait.

CASE IX.— *Simple fracture. Result imperfect.*

Catherine Bletzer, æt. 79, fell upon an icy side walk, in Dec., 1855, and was admitted to the Buffalo Hospital, on the 20th of the same month.

Her left thigh was broken through its neck. As she lay upon her bed the left side of the pelvis was drawn up, and her toes were everted. I directed pillows to be placed under her knees, and that she should remain upon her back, but that as soon as possible she should get up and walk about with crutches. She remained, however, nearly one year in bed.

Feb. 10, 1856. She left the hospital still unable to walk, and having only for a short time found herself able to sit up.

CASE X.— *Simple fracture. Result imperfect.*

R. W. Stockton, late surgeon in U. S. army, of North East, Pa., æt. 31, fell from a height, striking upon his feet and breaking the neck of the right femur.

His limb was laid on a straight single inclined plane, the foot being six inches higher than the hips. Extension was made by means of weights attached to bands passing over a pulley at the foot of the splint; the weight of the body accomplished the counter-extension. At the end of ten weeks he left the splint and began to use crutches.

Thirty-six years after the accident I examined the limb and found it shortened one inch and three-quarters. The union of the fragments was very firm and unyielding, and seemed to have been effected through the medium of bone. The foot and leg were slightly everted. Notwithstanding this amount of shortening, and although he used no extra heel to increase the length of the limb, he walked with only a moderate halt.

Case XI.—*Simple fracture. Result unknown.*

Isaac Lang, of Colden, Erie Co., N. Y., about 40 years old, broke his left thigh through its neck. I saw him Nov. 3, 1853, fourteen hours after the accident occurred. The messenger said that the attending surgeon, a very intelligent gentleman, called it a dislocation. Such, indeed, I found was his opinion, after my arrival at the house.

I found the hip much swollen; the thigh rotated outward, and shortened half an inch. Having placed him under the influence of chloroform crepitus was easily detected.

I applied my own straight splint with adhesive plaster extension.

He recovered in the usual time, but I have never learned the exact result.

Case XII.—*Simple fracture. Result imperfect.*

Mrs. F. W., of Buffalo, æt. 51. Feb. 3d, 1847, she slipped and fell upon an icy board, striking upon her left trocanter major. Dr. George Burwell was called. There was no shortening of the limb, but the foot was slightly everted. Yet the doctor apprehending a fracture had directed her to remain quiet in bed and upon her back, with her knees over a double inclined plane.

On the third day I visited Mrs. W. with Dr. Burwell. The limb was now shortened half an inch. No crepitus. We diagnosed a fracture of the neck of the femur. I left her in charge of Dr. Burwell. She remained in bed ten weeks.

May 29, 1854, seven years after the accident, I found her walking with a cane, and limping a good deal. The limb was shortened. The thigh and leg were slightly rotated outward. She had suffered more or less pain ever since the accident occurred, and at times the pain had been very great. She had been blistered and cupped; setons had been used, and cold baths. The latter alone had benefited her.

Sept. 10, 1856, nine years after the accident, I found her still walking with a cane, but able to walk without it. Her toes turn out and her thigh is shortened one inch. The flexors of the thigh are not sufficiently strong to lift the limb when the knee is bent.

Case XIII.—*Simple fracture. Result imperfect.*

Mrs. Dally, of Buffalo, æt. 67, fell, Oct. 19, 1853, from a trunk upon which she was standing, and struck upon her right hip.

I saw her soon after the accident occurred. The thigh was shortened half an inch. Toes everted. Hip excessively painful when moved; the pain was also accompanied with frequent spasmodic contractions of the muscles about the hip. No crepitus.

I laid her upon her back, with her knees over pillows, so as to elevate them very slightly. I supported the foot also in such a manner as to prevent eversion of the toes. She was directed to leave her bed as soon as possible and to get about on crutches.

Oct. 22, 1854. She walked with a cane, but not without limping. The thigh was shortened one inch.

Case XIV.—*Simple fracture. Result imperfect.*

John Sheenan, æt. 39, a United States soldier, received in battle a blow upon the left trocanter major, from the but end of a musket, fracturing the neck of the femur, probably without the capsule.

He was confined in a long straight splint six weeks.

Six years after the fracture, I found the thigh shortened two inches, and the toes slightly turned out. In rotating the limb the trocanter major moved upon a very short radius. There was considerable motion at the seat of fracture, and he was unable to walk unless the fragments were in a certain position.

Drs. Pride and Boardman, of Buffalo, were present and confirmed the results of this examination.

CASE XV.—*Simple fracture, within the capsule. Non-union of fragments, &c.*

Christophe Nirlear, a German emigrant, æt. 57. While in Montreal, C. E., he fell ten feet upon the wharf, breaking his left femur through its neck. In his fall he struck first on his feet and then pitched forward. He was taken to a hospital, the surgeons of which probably supposed it to be a dislocation. They pulled violently upon it when he was first admitted, and they repeated the same at a subsequent period, but they did not dress the limb with splints or apply any permanent extension.

Dec. 11, 1853, three years after the accident occurred, he applied to me at the Buffalo Medical College Dispensary, for advice and relief.

I found the thigh shortened one inch and a half. When standing the thigh was slightly rotated outward, but it was not at all when he was lying on his back. I could feel the trocanter major, and the neck of the bone leading from it distinctly; the neck lying upon the back of the pelvis, and terminating abruptly as if broken off close to the head. The head could not be felt. The broken neck seemed to play upon the back of the pelvis as if a new joint was formed at that point, but I could not feel any thing like a socket. The end of the bone was smooth and round. He walked tolerably well with the aid of a cane.

CASE XVI.—*Simple fracture. Result imperfect.*

Mrs. Wadsworth, of Buffalo, æt. 51, fell and broke the neck of the right femur, in the year 1841. Drs. Trowbridge and Winnie in attendance. No splints were used. She was not able to walk under six months.

In 1848, seven years after the accident, I found the thigh shortened three quarters of an inch.

CASE XVII.—*Simple fracture. Death.*

Dec. 27, 1851, L. F. T., of Buffalo, æt. 42, fell upon a slippery sidewalk, breaking the neck of the left femur. Mr. T. weighed about two hundred pounds, and he fell with his whole weight upon his left trocanter. He was unable to get up or to walk.

Dr. George Burwell was first called, but at his request I was invited to see Mr. T. on the same day, and to take charge of him.

The toe and knee of the left leg were turned out and could not be turned in except by great violence, and by the infliction of great pain. The limb was shortened from one quarter to one half an inch. Much pain in the groin and along the whole length of the limb. No crepitus. Drs. Sprague and Burwell concurred with me in the opinion that the femur was broken at its neck.

I applied a straight splint, with extension and counter-extension.

Feb. 7th, on the forty-second day, (six weeks,) I removed finally all of the dressings. On the 9th he sat up. On the 10th he walked about the room with the aid of crutches and a strong male assistant. On the 11th, also, he walked, in the same manner, across the room, came back to his bed, and as he settled his head upon his pillow, he died.

CASE XVIII. — *Simple fracture. Resulting in death.*

Mrs. Sarah Pattison, of Buffalo, æt. 76, slipped and fell on her hip breaking the neck of the femur, Aug. 26, 1846.

Dr. Caleb Austin, of Buffalo, was in attendance, and subsequently, Dr. Austin requested me to see the case with him. Mrs. Pattison had been for some time in feeble health, but at the time of the accident, she was able to walk out.

Her limb was laid for a time over a double inclined plane, but it was not confined rigidly to that posture. She recovered from the accident so far as to walk, with considerable assistance; but her health had suffered greatly from the shock and from the confinement, and she died a little less than four months after the accident occurred.

CASE XIX. — *Simple fracture within the capsule. Result imperfect. Prosecution for alleged malpractice.*

The following report of the trial to which this case gave rise, was written by myself, for publication, in 1849, and contains all of the essential facts:

"*Prosecution for alleged Malpractice.* — Supreme Court — John C. Basset *vs.* John B. Collins and Anthony Barney.

"In the fall of 1843, John C. Basset, of Independence, Allegany county, aged 48, then in good health but corpulent, was injured by the upsetting of his wagon and the falling of a box, as was believed, upon his thigh. He was carried into a public house, in Woodhull, and there attended by Drs. Reed and Carey. After a careful and complete examination by measuring, etc., they concluded that Mr. Basset had only received a severe bruise.

"He remained two weeks under their care and was then taken home in a bed. Four weeks after the accident, Drs. Collins and Barney were called in, as the left leg was now said to be shortened and turned out. These gentlemen made an examination and found the leg in the following condition: shortened an inch and a half; the toes turned out and could not be turned in; the left heel corresponding to the hollow of the right foot; a bunch in the groin like the head of the femur.

"They decided that it was a dislocation of the head of the femur upon the pubis, and with pulleys properly adjusted and carefully operated upon, proceeded to attempt its reduction.

"After two or three minutes' extension and counter-extension, a sound was heard, and a sensation felt by nearly all who were assisting, which was by them described as the sound and sensation usually produced when a dislocation is reduced. The patient was now released from the pulleys and made to get up. The limb was of its original length, and in its natural position, and the tumor in the groin had disappeared. The patient was again laid upon the bed and dismissed as cured.

"It however appeared in testimony, that a few days after, it was again shortened and turned out; but it does not appear that these facts came to the knowledge of the defendants. It also appeared that the plaintiff did not get the use of his limb so as to be able to dispense with crutches or a cane, in one or two years.

"The limb is now shortened an inch and a half, and moderately turned out; but the motions of the joint are free, and the plaintiff walks with a very slight halt and without inconvenience.

"Drs. Collins and Barney were prosecuted, and the case was tried in January, 1848, before Judge Marvin, but the Jury having disagreed, it was again tried before Judge Mullett, in the Circuit of the Supreme Court, held in August, 1848, in the town of Angelica, Allegany Co., N. Y.

"In the first trial the plaintiff charged that the limb was sound when the defendants took hold of it with the pulleys, and that they then fractured it through the neck and without the capsule.

"In the last trial this was not claimed, but it was alledged that the original accident was probably a fracture without the capsule, and without displacement; that when examined by Drs. Collins and Barney, a displacement had occurred, and that the defendants were chargeable with criminal negligence or ignorance, in not discovering that it was a fracture; and consequently for subjecting the plaintiff to the useless pain of extension with the pulleys, and in not applying subsequently a retentive apparatus, since through this omission the plaintiff had a shortened and crooked leg.

"On the defence it was admitted that the original accident was a fracture without displacement; but that it was *within* the capsule and near the head of the bone; that its being within the capsule and near the head could alone satisfactorily account for the "bunch" in the groin, which disappeared with the reduction, and for the slowness of the subsequent restoration of the limb. It was claimed, also, that the signs described by the witnesses were the ordinary signs of a dislocation upon the pubis, and would be likely to deceive the most skillful surgeon; that several eminent surgeons had mistaken fractures of the thigh for dislocations; that the extension with the pulleys did him no permanent injury; that the subsequent treatment pursued by the patient in this case, viz., keeping his bed for a few days and then getting about on crutches, would have been the proper treatment had the exact nature of the accident been fully known; and finally, that the patient had as good a limb as can ordinarily be expected in this fracture under the most skillful management.

"The examination of the numerous witnesses having closed, the jury were addressed by the distinguished gentlemen, Messrs. Doolittle and Skinner, for the defendants; and the Hon. Judge followed with a most pungent and impressive charge, in which the jury were instructed to disregard all mere appeals to their prejudices, and especially to reject that counsel which would advise them to look upon the medical profession as an *oppressive and aristocratic monopoly*, and to decide the case upon the facts as drawn from the witnesses on the stand.

"The jury retired, and in a few minutes returned a verdict for the defendants.

"The defendants in this case, were men who have long practiced both medicine and surgery in the county of Alleghany: Dr. Barney, at Independence, and Dr. Collins, at Alfred, and they both occupy a high position in the estimation of the public, as men of skill and worth. Dr. Collins was for some years an active member of the state legislature, and it is gratifying to know that in the mind of the Hon. Judge, as well as of the intelligent jury, they received a full and unqualified acquittal from the charge of any degree of negligence or unskillfulness.

"The medical witnesses examined on the part of the plaintiff, were Drs. John Read, of Woodhull, John R. Hartshorn, of Alfred, John H. Thorp, of Independence, and Charles D. Robinson, of Almond, all of Allegany Co., and Dr. Abijah Case, of Howard, Steuben Co.

"On the part of the defense, were Drs. Erastus Willard, of Friendship, Richard Charles, of Angelica, Randall Reed, of Amity, George B. Jones, of Scio, Lorenzo Dana, of Friendship, and Walter Wallace, of Angelica, all of Allegany Co., and Dr. Frank H. Hamilton, of Buffalo, Erie Co."

CASES XX AND XXI.— *Compound, comminuted fracture of both thighs complicated with fracture of the pelvis. Resulting in death after a few hours.*

John O'Keef, aged about 45 years.

(See Case VIII of Fractures of Ossa Innominata.)

THROUGH TROCANTER MAJOR.

CASE XXII.— *Simple fracture. Fragment displaced.*

James Redick, a traveling showman, æt. 23, fell, in Aug., 1848, from a high wagon, striking upon his left hip. When he got upon his feet he was unable to walk. Dr. Charles Wilcox was employed. Two weeks after the injury was received, I was requested to see him. I found the trocanter major carried upward toward the crest of the ilium, half an inch, and also slightly sent in; but the thigh was not shortened. There was no crepitus.

Oct. 11, 1848, I examined the patient and found no change in the position of the fragment. The functions of the limb were, however, almost completely restored.

CASE XXIII.— *Fracture complicated with other injuries. Death on the seventh day.*

Wm. Williams, of Buffalo, æt. 45, fell through a scuttle in a carriage house, Nov. 21, 1853, breaking the os frontis, and displacing the ossa nasi, and also breaking the right thigh through the trocanter major.

He was seen by Dr. James P. White and myself. Crepitus was very distinct in the broken trocanter. Whether the neck of the femur was broken or not we could not determine.

Mr. Williams died on the seventh day from the injuries to his brain.

BELOW TROCANTER MAJOR.—(UPPER THIRD.)

CASE XXIV.—*Simple fracture. Result nearly perfect.*

Martha Barker, æt. 7, was run over by a loaded wagon, Aug. 2, 1852, breaking her thigh through its upper third.

Dr. Leech was immediately called, and dressed the limb temporarily; on the following day he sent for me.

Assisted by Dr. Leech, I applied my own straight splint, with gutta percha side splints. We used also the adhesive plaster straps for extension.

Sept. 3d, the splints were finally removed. Limb perfectly straight, and shortened one quarter of an inch.

Sept. 10th, 1856, four years after the accident, we could not discover where the fracture occurred.

CASE XXV.—*Simple oblique fracture. Union delayed. Result imperfect.*

Sidney Allen, of Rochester, aged about 30 years, was thrown from a carriage, May 16, 1840, breaking his right femur, just below the trocanter major. Direction of fracture, obliquely downward and backward. He was also severely stunned by the fall.

I dressed the limb with Gibson's modification of Hagedorn's splint, making counter-extension, however, by means of a perineal band. I applied also, lateral splints with bandages, &c., and made extension with a silk lined gaitre. At the end of fourteen days, slight ulceration had occurred upon the instep, and the perineum was very intolerant of pressure. I removed therefore the straight splint and applied Bowen's splint. During the six first weeks I drew off his urine daily with a catheter, the bladder having for this time lost the power of expulsion of its contents. Bony union was delayed until about the tenth week, and before it was accomplished it became necessary to place him again upon the straight splint.

The thigh is shortened three quarters of an inch, and he limps very badly.

CASE XXVI.—*Simple fracture. Result imperfect.*

Gilbert Bogart, M. D., of Livingston Co., N. Y., fractured his right femur in the year 1845. The fracture was oblique, and about three inches below the trocanter major.

Dr. C. C. Chaffee, late Professor of Anatomy at Pittsfield Med. College, was requested to take charge of the limb, and, at the instance of Dr. Bogart himself, he treated the case throughout with a double inclined plane.

I saw Dr. Bogart at my office, May 12, 1847, and found the thigh shortened one inch and a half, but firm and nearly as useful as before.

CASE XXVII. — *Simple fracture. Result imperfect.*

E. Richardson, æt. 42. Simple fracture through the upper third. (I have no record of the name or residence of the surgeon.)

Four years after, I found the thigh shortened three quarters of an inch, and slightly bent at the seat of fracture.

CASE XXVIII. — *Simple fracture. Twice broken at same point. Result imperfect.*

Morris Cassady, æt. 9, fractured his right femur through its upper third. Surgeon Shields, of Ireland, treated the fracture each time.

Thirty-one years after I find the thigh shortened one inch and a half. The point where the fragments overlap can be distinctly felt. He walks with only a slight halt.

CASE XXIX. — *Simple fracture. Result imperfect.*

Antonio Gode, of New York, æt. 55, fractured the thigh through its upper third. He was treated at the New York City Hospital, with straight splints and extension. The extension was made with adhesive plasters.

June 30th, 1852. When I examined the thigh, six weeks after the fracture had occurred, it was united with a shortening of three quarters of an inch. The patient says the same thigh had been broken before, at the same point, and that it shortened some at that time, but that it is shorter now than before.

CASE XXX. — *Simple fracture. Result imperfect.*

John Cartwright, of Tonawanda, æt. 49, fractured his femur through its upper third.

Dr. Locke, of Tonawanda, had charge of the case. He laid the limb upon a double inclined plane.

Seven weeks after the fracture had occurred, I examined the limb and found it united with a shortening of half an inch.

Case XXXI. — *Simple fracture. Result imperfect.*

John Moffat, of Buffalo, about one year old, fractured the right femur near the lower end of the upper third, by a fall from his bed.

Dr. Wilcox in attendance. The lad was very fat, and the application of splints was rendered exceedingly difficult. No extension or counter-extension was used, nor a double inclined plane, but lateral splints were applied with as much care and diligence as possible.

One year after the accident, April 15th, 1852, I found the bone slightly bent forward at the seat of fracture, and when he walked the toes were inclined to turn inward. I could not detect any shortening of the limb.

Case XXXII. — *Simple fracture. Result imperfect.*

John Redman, of Buffalo, æt. 40, was admitted to the hospital with a fracture of the right femur near its upper end, Aug. 8, 1849.

The fracture was simple and oblique. On the following day I applied a paste bandage to the leg and foot.

On the 10th I applied side splints and secured the extending and counter-extending bands to a straight splint.

With only slight modifications, these means were continued until the 26th of August, a period of eighteen days, when a double inclined plane was substituted for the straight splint.

Sept. 11, all dressings were removed, and the bones were found to be united with a shortening of half an inch.

Case XXXIII.— *Simple fracture. Result imperfect.*

William Murphy, æt. 13, fell from a pile of boards, Oct. 28, 1855, fracturing the left femur at the lower end of the upper third. He fell upon his side. Fracture simple.

Dr. John Boardman, one of the hospital surgeons, dressed the leg at my request. He applied Potter's straight splint, making extension with adhesive straps, and counter-extension with a perineal band. A roller was applied to the whole limb, and also four lateral splints to the thigh, made of binders board.

I subsequently took charge of the case, and although I examined the limb daily, and took great pains by renewing and re-tightening the dressings, to render the cure as perfect as possible, yet the bone united with a shortening of one inch. The axis of the limb was, however, as nearly straight as this amount of overlapping would permit.

January 5th, 1856, ten weeks after the accident, he was still walking on crutches.

Case XXXIV.—*Simple fracture. Result imperfect.*

Almon Carpenter, of Buffalo, æt. 5, was struck by a heavy gate, Aug. 16, 1852, breakirg his left thigh near the lower end of the upper third.

On the same day I applied a straight splint, with side splints.

Sept. 13th, four weeks after the accident occurred, I removed all dressings. The fragments were united, but with an overlapping of three quarters of an inch. Limb straight.

I did not reapply the splints. At the end of eight weeks he was walking, and without any perceptible halt.

Case XXXV.—*Simple fracture. Result imperfect.*

Mrs. S. C. P., of Buffalo, about 55 years old, fell down a flight of steps, Feb. 24, 1853, breaking her thigh near the lower end of the upper third. Dr. Josiah Trowbridge and myself in attendance.

I dressed the limb with Welch's splint, using it at first as a double inclined plane. On the seventh day I converted it into a straight splint, and laid her upon a fracture bed. I used adhesive plasters for extension.

April 7th, I removed all dressings. Limb was found to be straight, but shortened half an inch. The knee and ankle remained very stiff, and lame for many months, but she was at length able to walk as well as before.

Case XXXVI.—*Simple fracture. Result imperfect.*

"Mrs. E. Howard, of Buffalo, æt. 47, was injured by the upsetting of her sleigh, the sleigh falling upon her left side, Dec. 24, 1854. Dr. Leech, of Buffalo, was called, but on the same day requested me to take charge of the case with him.

"I found her a feeble, dropsical woman. The left thigh was broken at its neck. The whole limb was greatly swollen and exquisitely tender. The toes were everted strongly, and could not be moved. The limb was shortened one inch. The muscles were frequently contracted spasmodically. We gave her chloroform and then detected a distinct crepitus.

"She was laid upon her back, in bed, with her knees over a couple of feather pillows. The foot was supported by bolsters. On the following day I laid the limb over Welch's double inclined plane, using great care to cushion and support the limb. My purpose, in laying the limb on this splint, was to support and steady it in such a manner as to save her from the great pain which she suffered whenever the limb was in any way disturbed. Nothing, however, in this respect was gained, and I soon changed it for my own straight splint.

"Jan. 5, 1855. She still continued to suffer greatly and I laid aside all apparatus, and placed her on one of Coolie's fracture beds."

The above is the record of this case which I had prepared for publication, when, Sept. 8, 1856, I visited Mrs. Howard at her house, and found her confined to her bed with ascites, and other chronic maladies. She permitted me to examine her broken limb, when, for the first time, I found unequivocal evidence that the fracture had occurred through the *shaft* of the femur instead of through the *neck*. The dropsical and inflammatory swelling had now entirely disappeared from this part of the limb, and the bone could be distinctly felt bent forward at the seat of fracture, and irregular in its form. This part of the limb also remained tender. The limb was shortened one inch. She has never walked without a crutch or cane since the accident. Her knee, she says, has been the greatest source of lameness.

It is due to myself to say, that I was in some doubt during the first six or seven days, as to whether it might not be a fracture of the shaft rather than of the neck. The swelling of the limb was always such that, even when she was under the influence of chloroform, a satisfactory diagnosis was difficult: but after several careful examinations I felt quite certain that the fracture was through the neck, and so recorded it.

Case XXXVII.—*Simple fracture. Re-fractured at same point. Result imperfect.*

James Leacock, æt. 29, fractured his thigh at the lower end of the upper third, by a fall from a height upon the ground. Dr. Parsons, of Portageville, Livingston Co., N. Y., was employed. He treated the limb with a straight splint, and in abont six or seven weeks he was able to leave his bed, with the limb shortened about three quarters af an inch. This is the statement made to me by Leacock himself.

Dec. 27, 1848, about nine months after the first fracture, he fell upon his knee with moderate force, and re-fractured the limb at the same point.

On the same day he was admitted to the Buffalo Hospital of the Sisters of Charity, and I applied my own straight splint, with lateral splints, &c. The limb was easily brought down so as to be of the same length as before the last fracture occurred: that is, to within three quarters of an inch of the length of the opposite limb.

On the 29th vesications had occurred upon the top of the ankle from the pressure of the gaitre. The extending bands were loosened and the gaitre re-adjusted. Notwithstanding great care was used to prevent this event, a slough occurred above the heel, on the 3d of June. From this time to Feb. 16, we were compelled, by his constant complaints, and by his restlessness, to change the mode of extension and support, often. The extension was made, from above the ankle, from above the ham, from above the knee, alternately, or at the same time, with adhesive plasters and cotton, or flannel rollers; once, for a short time, the double inclined plane was used.

Feb. 16, fifty-two days from the commencement of the treatment, we found the bone united, and the limb of the same length as before: that is, shortened three quarters of an inch.

In this case there were present the usual sources of difficulty, to which were superadded an exceedingly irritable temperament, and the previous occurrence of fracture at the same point.

Case XXXVIII.—*Simple fracture: occurring four times consecutively at the same point. Very great shortening.*

J. H. Frick, of Williamsville, Erie Co., N. Y., sent for me, June 18, 1849, having broken his left femur sixteen days before I saw him, by jumping from a wagon. The fracture was at the lower end of the upper third.

This is a remarkable case. When only 16 years of age he first broke this thigh by a fall on the sidewalk. A Thompsonian, who was living at Williamsville, dressed it with only short, side splints. Union was delayed, and at the end of nine weeks he sent for a man by the name of Smith, who had once been a blacksmith, but who was now a Doctor Smith, and set bones instead of horse shoes. Smith bound the limb with leather, and after five weeks more union occurred. One year from this time, he broke it again while carrying a moderate weight in his hands. Smith dressed the leg this time, also, and in the same manner. The bone united in about two weeks. At the age of 22 he broke it again. So Smith said. And Smith treated it successfully again. Each time the fracture was at the same point. On this last occasion he employed an experienced surgeon, Dr. Van Pelt, of Williamsville. Dr. Van Pelt had treated it with a straight splint, and the bone seemed already firm on the sixteenth day. The limb was shortened, according to measurements made by Drs. Van Pelt, Hawley and Mr. Otis, medical student, with myself, three inches and seven-eighths. It was, however, as perfect in all respects as it was before the last fracture.

CASE XXXIX.—*Simple fracture, complicated with other injuries, resulting in death.*

Thomas Ryan, of Buffalo, æt. 14, was injured by the cars, at Syracuse, breaking his left thigh just below the trocanter minor, severely bruising the right leg, and rendering necessary the amputation of the right arm above the elbow joint. The amputation was made, and the limbs dressed, by a surgeon in Syracuse.

About three weeks after the accident occurred, he was brought to Buffalo, and I was requested to see him. The stump had not yet healed. There was a large abscess discharging freely in the vicinity of the right knee. The broken thigh was resting upon Day's splint, the splint being extended. Extension was made with adhesive plasters, but there was no counter-extending band. It was neatly bandaged and carefully adapted side splints supported the fragments. No union existed between the ends of the fragments, and they were overlapped three quarters of an inch. I re-applied the same dressings carefully, so far as it was nece.sary to do so, and dressed the wounds. On the following day an empiric was employed, and in about one week he died.

CASES XL AND XLI.—*Compound, comminuted fracture of both thighs. Resulting in death.*

An Irish laborer was crushed between two cars in a collision which occurred on the New York Central Railroad, May 16, 1854, breaking both thighs near their upper ends. The fractures were compound and comminuted.

I sent him immediately to the Buffalo Hospital, where he survived the accident only a few days, having never completely rallied from the shock.

(To be Continued.)

